# Metabolic reprogramming and signalling cross-talks in tumour–immune interaction: a system-level exploration

**DOI:** 10.1098/rsos.231574

**Published:** 2024-03-13

**Authors:** Mudita Shukla, Rupa Bhowmick, Piyali Ganguli, Ram Rup Sarkar

**Affiliations:** ^1^ Chemical Engineering and Process Development Division, CSIR-National Chemical Laboratory, Pune, Maharashtra, India; ^2^ Academy of Scientific & Innovative Research (AcSIR), Ghaziabad, Uttar Pradesh 201002, India

**Keywords:** tumour–immune interaction, metabolic reprogramming, signalling-metabolic cross-talks, system modelling

## Abstract

Tumour-immune microenvironment (TIME) is pivotal in tumour progression and immunoediting. Within TIME, immune cells undergo metabolic adjustments impacting nutrient supply and the anti-tumour immune response. Metabolic reprogramming emerges as a promising approach to revert the immune response towards a pro-inflammatory state and conquer tumour dominance. This study proposes immunomodulatory mechanisms based on metabolic reprogramming and employs the regulatory flux balance analysis modelling approach, which integrates signalling, metabolism and regulatory processes. For the first time, a comprehensive system-level model is constructed to capture signalling and metabolic cross-talks during tumour–immune interaction and regulatory constraints are incorporated by considering the time lag between them. The model analysis identifies novel features to enhance the immune response while suppressing tumour activity. Particularly, altering the exchange of succinate and oxaloacetate between glioma and macrophage enhances the pro-inflammatory response of immune cells. Inhibition of glutamate uptake in T-cells disrupts the antioxidant mechanism of glioma and reprograms metabolism. Metabolic reprogramming through adenosine monophosphate (AMP)-activated protein kinase (AMPK), coupled with glutamate uptake inhibition, was identified as the most impactful combination to restore T-cell function. A comprehensive understanding of metabolism and gene regulation represents a favourable approach to promote immune cell recovery from tumour dominance.

## 1. Introduction

Tumour-immune microenvironment (TIME) evolves as a mini-ecosystem, leading to an alteration of both tumour and immune cell metabolism [1]. Immune cells undergo metabolic adjustments to endure and function effectively in response to rapidly proliferating tumour cells. Metabolic characteristics, such as glycolysis, fatty acid production and glutaminolysis of T-cells and natural killer cells, are impaired [2]. In a nutrient-deprived and hypoxic environment, immune cells become exhausted and lose their ability to produce cytokines and kill tumour cells. Moreover, metabolites produced by tumour cells, such as glutamate and lactate, inhibit immune cell function and promote immune suppression [3]. To promote an anti-tumour immune response, it is necessary to boost the nutrient availability of immune cells while concurrently reducing the production of metabolites like lactate.

Numerous targets were explored previously to improve the anti-tumour immune response. For example, glycolysis in immune cells was restored by blocking programmed death-1 and cytotoxic T-lymphocyte-associated antigen-4, over-activating the mammalian target of rapamycin complex 1 (mTORC1) pathway and inhibiting glucose availability to tumour cells [4–6]. Targeting glucose and lactate transporters, along with tumour-produced metabolites such as tryptophan, adenosine and arginase, has also been reported to strengthen the immune response [7,8]. In addition to these targets in tumour cells, metabolic alterations in immune cells were also studied previously. Evidence suggests that immunosuppression caused by glutamate release from glioma can be mitigated by regulating glutamate levels in T-cells [9,10]. In addition, succinate regulating the pro-inflammatory response of macrophages was also studied previously [11]. Although these studies shed light on the role of metabolism in regulating the immune response, there remains a significant gap in understanding how metabolic reprogramming in immune cells can enhance the immune response specifically against glioma, which is the most aggressive heterogeneous tumour with a low survival rate. The effectiveness of previously studied targets against glioma is limited and requires additional investigation. Glioma originates from glial cells, whose basic function is to support neuronal activity. These are highly metabolically active cells. Astrocytes are the most abundant glial cells, accounting for about 20–40% of the brain cells [12] and are the source of origin of astrocytoma and grade IV glioma [13]. In the present work, we study the astrocyte as the normal glial cell to understand its signalling and metabolic properties and to comparatively discuss the changes occurring in glioma.

*In silico* modelling approaches have enabled the large-scale analysis of intricate systems such as tumour–immune interactions to understand the underlying mechanisms. A couple of mathematical models were proposed to understand regulatory mechanisms governing glioma–immune interactions. For example, dynamics between glioma, macrophage, cytotoxic T-cell, transforming growth factor-beta (TGF-β) and interferon-gamma were developed using a spatiotemporal modelling approach [14]. The growth, suppression and elimination of glioma were measured by monitoring T-cell and macrophage-secreted cytokines and chemokines [15]. These models primarily focus on signalling pathways and overlook the effects of metabolic stress generated due to nutrient competition. Although an integrated model of signalling and metabolic pathways was developed for glioma with a focus only on epidermal growth factor receptor-driven information flow [16], a comprehensive integrated model of signalling and metabolic pathways for glioma, T-cell and macrophage, along with cross-talks between these pathways within and across cell types has not been studied previously.

Hence, by reconstructing individual signalling and metabolic maps of each cell and implementation of cross-talks between and within cells, a system-level model consisting of glioma, T-cell and macrophage is developed. The uniqueness of this model lies in its novel approach to understand tumour–immune interactions. The notable feature of the model is its ability to capture signalling and metabolic regulation at diverse time scales, which is achieved through the regulatory flux balance analysis (rFBA) approach.

The model is developed to predict the targets through which anti-inflammatory immune responses can be reverted to pro-inflammatory state against glioma. Through model simulations, certain unexplored phenomena are investigated. While glutamate inhibiting the T-cell response is known, the modulation of the antioxidant defence mechanism of glioma and the activation of T-cell response by blocking glutamate uptake to T-cell are yet to be explored. Targeting antioxidant mechanisms in glioma presents a promising approach for preventing tumour growth while also enhancing anti-tumour immunity. In addition, the combined effect of succinate and oxaloacetate (OAA) in shifting the metabolic properties of macrophage from an anti-inflammatory to a pro-inflammatory state remains unexplored. The dynamic regulation of reprogrammed metabolism on T-cell and macrophage response leading to differential type 1 T helper (Th1), type 2 T helper (Th2), T-regulatory (Treg), pro-inflammatory macrophage (M1) and anti-inflammatory macrophage (M2) phenotypes is largely unexplored. The successful implementation of metabolic reprogramming of immune cells through signalling metabolic cross-talks is crucial, and it is challenging to co-culture and capture such scenarios through *in vitro* experiments. Discovering synergistic combinations of signalling and metabolic targets that might improve anti-tumour immunity can be a promising future therapeutic approach. It is only feasible to comprehend the cross-talks between glioma, T-cell and macrophage through dynamic metabolic and signalling interactions using mathematical modelling.

## 2. Methods

### 2.1. Reconstruction of metabolic and signalling network

Cell-specific differentially expressed genes were identified independently from two RNASeq tissue-specific datasets for glioma (accession no. GSE79338) and macrophage (accession no. GSE115397). Genes with a *p*-value of <0.05 were marked as differentially expressed using the edgeR and subsequently used for pathway enrichment analysis in the GeneCodis tool [17,18]. Significantly enriched (*p*‐value <0.05) signalling and metabolic pathways related to the glioma study were filtered for the network reconstruction. The T-cell model was built by acquiring pathways from the literature.

Literature and various databases were queried to collate the information on metabolic and signalling pathways. The reconstructed network consists of 28 metabolic pathways and 33 signalling pathways (electronic supplementary material, tables S1 and S2 of file S1). The signalling network includes 192 signalling molecules (electronic supplementary material, tables S3 and S4 of file S1), and the metabolic network includes 326 reactions and 357 metabolites in the model (electronic supplementary material, file S2).

**Figure 1.**
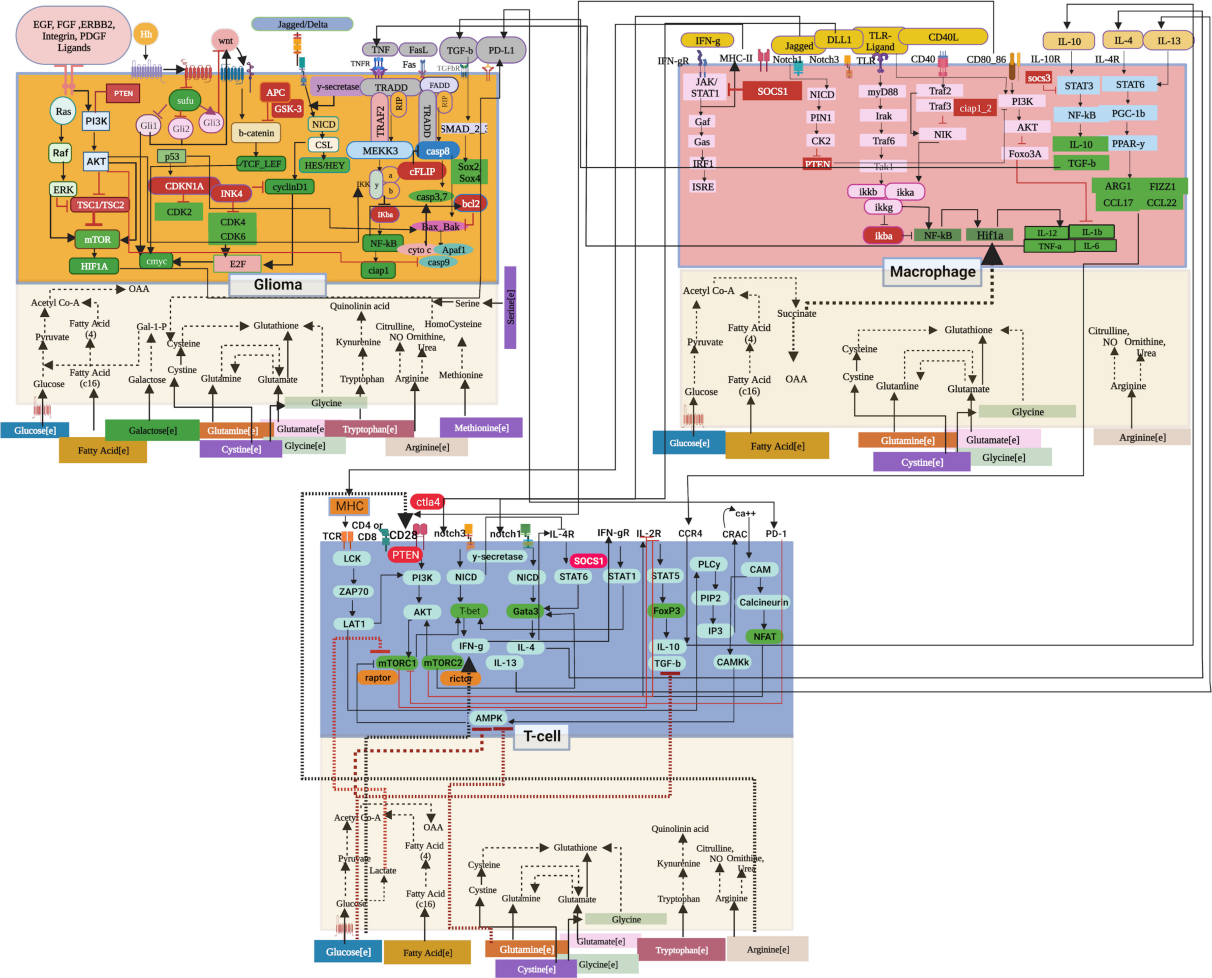
Pathway diagram of the reconstructed signalling and metabolic interaction network for glioma, T-cell and macrophage in TIME. Signalling and metabolic systems are colour segregated for each cell (glioma, T-cell and macrophage). Output signalling molecules are represented in green colour and inhibitors in red. Interconnections between signalling molecules and metabolites are represented with dotted lines if they are activators (black) and inhibitors (red).

A detailed pathway map depicting the cross-talk between glioma, T-cell and macrophage (figure 1) was created, which was drawn using the Biorender tool [https://biorender.com]. Cell-specific pathways and their interactions were separated by different coloured boxes. A summarized list of cell-specific complete network statistics is provided in electronic supplementary material, table S5 of file S1.

### 2.2. Integration of metabolic and signalling networks

To investigate the interplay between signalling and metabolism, cross-talks of these pathways were curated from the literature and included in the model. Metabolites such as glucose, glutamine, fatty acid, arginine, lactate and succinate were previously reported as crucial players in regulating the expression of various signalling molecules, which, in turn, regulate the differentiation of T-cell and macrophage [1,6,19]. For example, the activity of mTORC1_T was regulated by glucose, glutamine, lactic acid and arginine [20]. On the other hand, mTORC2_T (mammalian target of rapamycin complex 2) activity was regulated by arginine in the model (figure 1). These molecules are central to the development of T-cell into Th1 and Th2 cells [21]. The effect of glucose and glutamine on mTORC1_T was integrated through AMPK_T in the model [22]. Typically, the impact of arginine on T-cell activation and proliferation is measured through CD3_T (cluster of differentiation 3) and CD28_T (cluster of differentiation 28) co-receptors of TCR (T-cell receptor) [23,24]. In the model, CD28_T was defined as a direct effector of arginine availability. Cell specific nodes were added with suffixes: _T (T-cell), _M1 (pro-inflammatory macrophage), _M2 (anti-inflammatory macrophage) and _C (glioma).

### 2.3. Model formulation

#### 2.3.1. Metabolic network: flux balance analysis

Flux balance analysis (FBA), the most popular constraint-based modelling approach, was adopted to model the metabolic network [25]. Its ability to perform without the requirement of detailed kinetic parameters makes it feasible to model large and complex networks. However, FBA determines fluxes at a steady state and limits its ability to capture dynamic changes in fluxes occurring over time. Additionally, FBA does not account for regulatory information, which is a crucial factor in influencing flux distributions [26]. FBA is considered as a mechanistic approach to modelling as it relies on the stoichiometric matrix and well-classified metabolic network.

In FBA, constraints are imposed in the form of mass balance and capacity. Mass balance constraints were represented as positive if the metabolite was produced and negative if the metabolite was consumed. The range of capacity constraint was defined as lower bounds (lb) and upper bound (ub). The rate of change in each metabolite (*X*) concentration at a steady state (equation (2.1))


(2.1) $$\frac{d}{dt}X_{\left\{m\;\times \;1\right\}}=S_{\left\{m\;\times \;n\right\}}\;\times \;v_{\left\{n\;\times \;1\right\}}=0$$

Reaction bounds:


$$\begin{array}{c} -1000<v_{\left\{n\times 1\right\}}<1000\;\;\;if\;a\;reaction\;is\;reversible\\ 0<v_{\left\{n\times 1\right\}}<1000\;\;\;\;\;if\;a\;reaction\;is\;irreversible\\ -1000<v_{\left\{n\times 1\right\}}<0\;\;\;\;if\;a\;reaction\;is\;irreversible \end{array}$$

where $S_{\left\{m\times n\right\}}$ is the stoichiometric matrix of 357 metabolites (m) and 326 reactions (n) (electronic supplementary material, file S2) and $v_{\left\{n\times 1\right\}}$ is the reaction flux.

An example model with steps of metabolic model formulation through FBA is represented in electronic supplementary material, figure S1 of file S1, and the source code is provided in electronic supplementary material, file S3.

##### 2.3.1.1. Selection of objective function

Targeting the tricarboxylic acid (TCA) cycle can be an attractive approach to treating glioma due to its interconnection with multiple metabolic pathways [27]. For instance, succinate helps in glioma stem cell maintenance. Its accumulation alters metabolic pathways such as central carbon metabolism [28,29]. On the other hand, OAA is known for its role in altering the bioenergetics of glioma [30]. Glutathione (GSH) is also reported to maintain the redox balance, and its depletion causes cell death in glioma [27]. Furthermore, the combined requirement of OAA, succinate and GSH in glioma growth is defined in the literature [30]. Therefore, these components were selected in the model as an objective function in terms of fluxes, designated as Growth_Glioma (equation (2.2)), to fulfil the growth and energy requirements of glioma.


(2.2) Growth_Glioma=OAA_c+GSH_c+succ_c

Here, ‘succ’ represents, succinate in glioma. ‘_c’ represents, ‘glioma’. The growth and energy requirements of astrocyte cells can be fulfilled by optimizing the adenosine triphosphate (ATP) synthase (equation (2.3)) of oxidative phosphorylation (OXPHOS) [30].


(2.3) $$ATP\;synthase=>ADP+Pi+4H=H_{2}O+ATP+3H$$

The objective functions were linearly optimized using the ‘Linprog’ function of MATLAB R2020. By defining two distinct objective functions, glioma- and astrocyte-specific metabolic components were optimized, and the behaviour of T-cell and macrophage was observed under the influence of glioma and astrocyte. The design of the constraint-based model allows flexibility in choosing the objective functions. While the current objective emphasizes the growth requirements of the cell, one can choose different objective functions that might highlight metabolic properties during other cellular requirements, such as energy demand, migratory behaviour and dormancy of the cell. Hence, changing the objective function might lead to different optimization scenarios that may or may not differ from the current optimization.

##### 2.3.1.2. Lower and upper bounds

Astrocyte and glioma-specific metabolic properties were obtained by assigning limited upper and lower bounds to particular reactions, such as the transport of fatty acid, cystine-glutamate antiporter (xCT), glutamate dehydrogenase, cytochrome c oxidase (complex IV) and pyruvate dehydrogenase (PYDH) (electronic supplementary material, table S6 of file S1).

### 2.3.2. Signalling network: Boolean analysis

The Boolean network modelling approach was adopted to capture the overall network dynamics of signalling processes [31]. It is important to capture the interconnection and interdependence of molecules within and between cells of the signalling network in order to understand the tumour–immune interactions. Boolean modelling approaches are best suited for large and complex networks, offering qualitative understanding of the model. However, they lack the ability to capture quantitative and dynamic variations in the model. Integrating Boolean with other modelling approaches such as constraint-based modelling approaches presents a promising solution to overcome these limitations [32]. Boolean-based approaches can be considered as phenomenological modelling approaches as the system can be tuned to suit the phenomenon based on the condition/context of the system. Logical equations connecting each molecule are provided in electronic supplementary material, table S7 of file S1. The signalling molecules without any upstream regulation were considered as input molecules, and a total of 34 input molecules were incorporated into the model. The initial states of input molecules were curated from the literature and defined in electronic supplementary material, table S8 of file S1. All the simulations were performed using a synchronous update scheme in MATLAB R2020, separately for astrocyte and glioma conditions until a steady state was achieved.

### 2.3.3. Integration of metabolic and signalling networks: rFBA

rFBA integrates regulatory information along with metabolic networks using Boolean / logical operators. This integration of transcriptional events together with metabolism helps to understand the influence of the regulatory network over metabolism and vice versa. It provides a more comprehensive understanding of biological systems. However, rFBA is also based on steady-state assumptions and does not account for dynamic temporal changes in gene expression [26]. Furthermore, the association of kinetic parameters together with stoichiometry in rFBA assigns it as a mechanistic approach to modelling. By incorporating a regulatory constraint into the FBA framework, glioma growth and the immune response in TIME can be predicted [33,34].

#### 2.3.3.1. Regulation of metabolism on signalling

Once signalling and metabolic networks were defined, the regulations between signalling molecules and metabolites were incorporated into the network and represented as Boolean / logical equations. To measure the effect of metabolism on signalling, metabolites were assigned with initial concentration and the rate of change of metabolites concentration was measured. The initial concentration of metabolites was defined as a basal physiological metabolite concentration at which the activation of signalling molecules occurred. The final concentration was selected beyond which T-cell and macrophage activity was modulated. The initial and final concentrations of these metabolites were extracted from the literature (electronic supplementary material, table S9 of file S1). Regulation of metabolism on signalling was implemented through regulatory rules (equations (2.4-9):

Regulatory Rules:


(2.4) CD28_T=CD80_86_M1&not(ctla4_T)∣[(Arginine_T)>0.02)]

(2.5) AMPK_T=CAMKK_T&[not((Glucose_T)>=0.5)&not((Glutamine_T)>=0.5)]

(2.6) mTORC1_T=AKT_T&raptor_T&not(AMPK_T)∣(AKT_T&raptor_T&not(PD_1_T))∣AKT_T&raptor_T&[not(Lactate_T>3)]

(2.7) TGF_b_T=FoxP3_T&[not(Glucose_T>=0.5)]

(2.8) HIF1A_M1=(Succinate_M1>=1)|NF_kB_M1

(2.9) IFNg_T=T_bet_T&(Glucose_T>=0.5)

Assuming that signalling and metabolism are required to initiate any immune response, logical rules connecting signalling and metabolism were represented with the ‘AND’ logical operator, except for the activation of HIF1A_M1 and CD28_T. The activation of HIF1A_M1 and CD28_T was illustrated with the ‘OR’ operator by considering that other signalling molecules within these pathways can also influence the expression of HIF1A_M1 and CD28_T and connecting them with the ‘AND’ operator would create bias. Using the OR operator allows for more flexibility in activating these pathways.

#### 2.3.3.2. Regulation of signalling on metabolism

A regulatory constraint was imposed on the gene product of metabolism to measure the effect of signalling on metabolism. In the model, regulation of the AMPK signalling molecule on fatty acid metabolism was implemented (equation (2.10)).


(2.10) RegulatoryRule:Pa_T=AMPK_T

The effect of ‘AMPK_T’ on the ‘transport of fatty acid for oxidation (Pa_T)’ in T-cells was measured.

*Time scales*. Different signalling and metabolic processes vary in response times; specific signalling and metabolic pathways may involve slower cascades of events, while others may transmit signals quickly, resulting in rapid cellular responses. Metabolic processes are fast and require shorter time steps. On the other hand, transcriptional regulation of signalling molecules through metabolites and vice versa, involving gene expression and protein synthesis, requires longer time steps [33,35]. Since it is challenging to account for the time delays of each molecule, the time delay was incorporated in the simulations so that the model accurately validates the characteristics of each cell. Several combinations of time intervals were explored and an optimal value at which the model accurately captures the cell-specific signalling and metabolic properties was achieved. In the model, metabolic processes were assigned shorter time intervals (*dt* = 0.07), and transcriptional regulations were assigned longer time intervals (*dt* = 1), indicating that metabolism is fast and requires less time to consume, while transcriptional regulations are slow and require more time to show a response. At the assigned time scale, cell-specific behaviour in glioma-dominant and normal astrocyte conditions were validated with pre-existing experiments and provided under the model validation section.

### 2.3.4. Model analysis and perturbation studies

Metabolic and signalling properties of astrocyte and glioma, as well as T-cell and macrophage in both astrocyte and glioma scenarios, were evaluated by optimizing two different objective functions. Additionally, the flux through specific reactions was restricted to obtain the cell-specific behaviour in both normal and glioma-dominant conditions. The obtained responses were then validated with the literature. To restore immune cell function from tumour dominance, the flow of metabolites between glioma and immune cells was altered. The impact of altered metabolites was measured on the overall metabolism of T-cells and macrophages. Subsequently, the effects of reprogrammed metabolism on differential expressions of T-cell (Th1, Th2 and Treg) and macrophage (M1 and M2) subtypes were evaluated. Lastly, metabolic reprogramming was performed by combining signalling and metabolic targets to improve immune response in the tumour microenvironment. The term ‘metabolic reprogramming in the model’ refers to modifications in the cell’s metabolism due to either perturbation of metabolites or regulation through signalling molecules. Perturbation was incorporated either by altering the stoichiometry of the exchange reactions or by varying the lower and upper bounds of certain reactions. Simulations were run for 2000 time steps with different time intervals assigned for signalling (*dt* = 1) and metabolism (*dt* = 0.07).

## 3. Results

### 3.1. Model validation

The present model incorporated significant known altered interactions of signalling and metabolic pathways in the context of tumour–immune regulation. Validation of signalling and metabolic properties was performed from publicly available datasets. The model dynamics appropriately captured the behaviour of astrocyte and glioma, as well as T-cell and macrophage in the astrocyte and glioma conditions.

#### 3.1.1. Validation of metabolic properties

**Figure 2.**
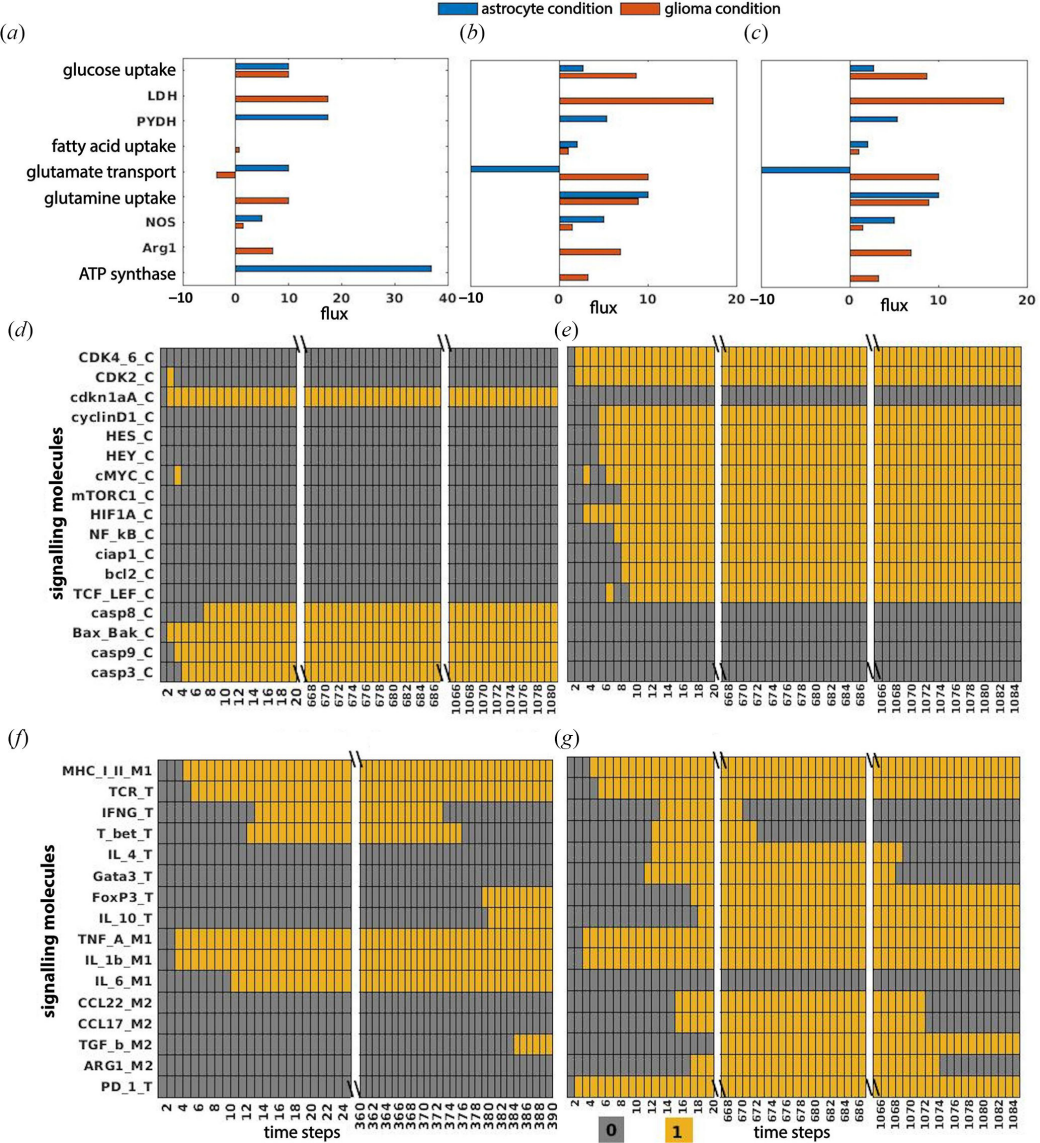
Validation of metabolic and signalling properties: flux distribution of selected metabolic reactions in: (*a*) glioma versus astrocyte, (*b*) T-cell and (*c*) macrophage in glioma versus astrocyte. Expression profile of selected output signalling molecules in: (*d*) astrocyte, (*e*) glioma, (*f*) T-cell and macrophage in astrocyte, (*g*) T-cell and macrophage in glioma-dominant conditions.

The metabolic properties of astrocytes and glioma were validated from the literature. The occurrences of astrocyte-specific metabolic properties, such as ATP synthesis through OXPHOS, elevated glutamate uptake and diminished fatty acid oxidation (FAO), were observed in the model and found to be consistent with the astrocytic behaviour [30,36]. Glioma-specific metabolic properties, such as glycolysis leading to lactate formation and downregulation of PYDH, were also observed [37]. Furthermore, the model exhibited higher flux through glutamine uptake in glioma compared with astrocytes, enhanced release of glutamate through xCT, higher flux through arginase, downregulation of OXPHOS and enhanced FAO [38,39], which are the characteristic metabolic properties of glioma and serve to validate the glioma behaviour within the model (figure 2*a*).

Next, metabolic characteristics of immune cells in astrocyte and glioma conditions were validated. Resting T-cells rely on OXPHOS and FAO, whereas activated T-cells depend on aerobic glycolysis to obtain energy [40,41]. Similarly, macrophages use distinct metabolic pathways for M1 and M2 activation and polarization. M1 macrophages depend on processes like anaerobic glycolysis, pentose phosphate pathway (PPP) and fatty acid biosynthesis, whereas M2 macrophages primarily depend on OXPHOS and support catabolic metabolism to sustain their metabolic requirements [42]. In the astrocyte condition, reduced uptake of glucose and increased FAO, downregulation of OXPHOS, increased uptake of glutamine, increased production of nitric oxide (NO) and release of glutamate indicate the occurrence of resting and immune effector type of metabolic properties. Astrocytes are surrounded by resting and activated phases of T-cells and macrophages [43,44]. The observed metabolic properties validate the occurrence of both resting and effector types of immune cells in the astrocyte condition.

In the glioma-dominant condition, comparing the metabolic profile of T-cell and macrophage with glioma showed that the distribution of important metabolites was skewed in favour of glioma growth (electronic supplementary material, figure S1 of file S1). Glucose availability becomes limited for the activated T-cells and macrophages, and they depend on alternative energy sources like fatty acid and glutamate to maintain cellular function [20,45]. Increased flux through arginase production, reduced flux through nitric oxide synthase (NOS) and decreased glutaminolysis (GLS) compared with the astrocyte condition indicate the downregulation of T-cell and macrophage [1,46] (figure 2*b,c*).

#### 3.1.2. Validation of signalling properties

The dynamics of signalling processes were validated by selecting 33 output signalling molecules (17 glioma, 8 T-cell and 8 macrophage-specific). The steady-state expression of glioma-specific output molecules was validated from the literature and RNASeq data obtained from The Cancer Genome Atlas (TGGA-https://portal.gdc.cancer.gov/). The expression of 60% proteins was accurately mapped with the ‘Fold Change (FC) value’ obtained from RNASeq data analysis. However, the expression of all output proteins from the model was matched with the literature (electronic supplementary material, table S10 and S11 of file S1). In glioma, cell cycle-dependent kinases (such as CDK2_C and CDK4_6_C) and tumour growth-promoting molecules (such as mTORC1_C, cMYC_C and NF-κB_C) were observed to be upregulated [47,48]. Signalling molecules of cell cycle arrest and apoptosis inhibition, such as cdkn1a_C, bcl-2_C and cIAP1_C, were downregulated in glioma and upregulated in astrocytes [49] (figure 2*d,e*).

Steady-state values of immune cell-specific signalling output molecules were qualitatively matched with the literature in glioma and normal astrocyte conditions (figure 2*f,g*). T-box transcription factor (T_bet_T), Gata3_T and FoxP3_T were selected as representatives of the Th1, Th2 and Treg responses of T-cells, respectively. HIF1A_M1 and ARG1_M2 are representatives of M1 and M2 responses of macrophages, respectively. MHC-linked Th1 response through the mTOR pathway was activated only for a few time steps in the glioma scenario [50]. Treg cells secreted factors such as FoxP3_T and TGF_b_T, which promote glioma expansion, were upregulated in the model [51]. Similarly, the downregulated M1 response and upregulated M2 response of macrophages in the model align with the reports of the presence of M2 macrophages in glioma progression [52]. Lower expression of T_bet_T (Th1) and higher expression of Gata3_T (Th2) further validate the glioma model [53]. The expression profile of the signalling molecules obtained in the model represents the first antigen encountered by immune cells against the complete development of glioma. All details related to signalling molecules are provided in the electronic supplementary material, table S3 of file S1.

### 3.2. Model analysis

#### 3.2.1. Metabolic reprogramming through modulation in the flow of succinate and oxaloacetate between glioma and macrophage

It was observed from the model that succinate and OAA regulate the metabolism of glioma and macrophage in TIME. Previous report suggests the exchanges of TCA cycle intermediates between glioma and macrophage [11]. The role of succinate in regulating the M1 response was also reported [54]. However, the exchange of OAA from macrophages and its significant role in shifting immune response towards a pro-inflammatory state were not studied previously. Recovery of the immune cell from the glioma dominance condition was created in the model by modulating the exchange of succinate and OAA from the macrophage and compared with the glioma-dominant condition. Through the model analysis, it was observed that the release and uptake of these TCA cycle intermediates between glioma and macrophage were coupled with each other and played an important role in reverting the immune response from anti-inflammatory to pro-inflammatory.

**Figure 3.**
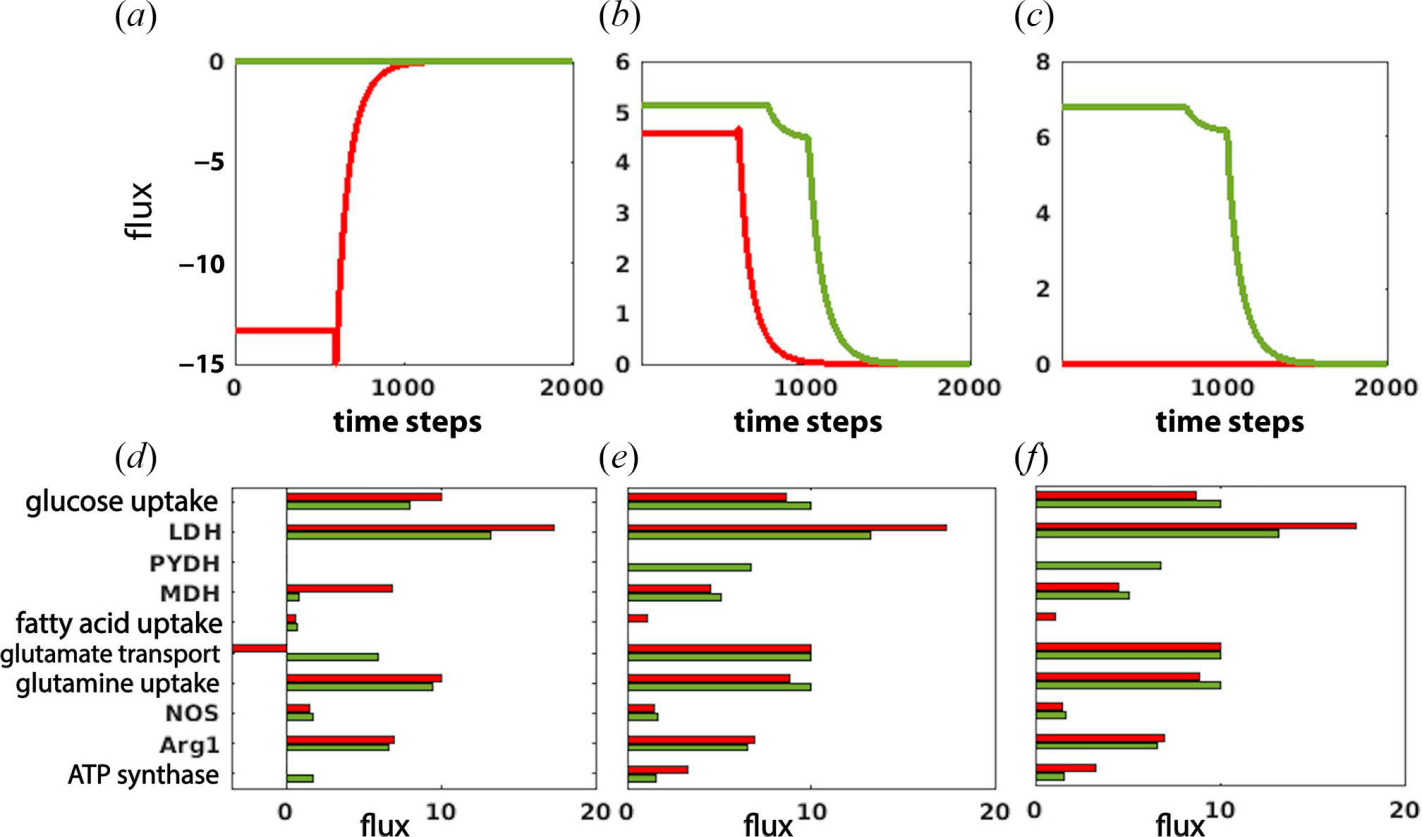
Metabolic reprogramming resulted from modulation in flow of succinate and OAA through macrophage: (*a*) exchange of OAA, (*b*) malate dehydrogenase (MDH), (*c*) PYDH in macrophage. Reprogrammed metabolic properties in: (*d*) glioma, (*e*) T-cell and (*f*) macrophage. Recovery of immune cells from glioma dominance (green) is obtained after this perturbation and compared with the glioma-dominant condition (red).

A temporal analysis of the flux distribution in glioma and macrophage showed an increase in the exchange of OAA from macrophage in the glioma dominant condition, and no exchange was observed during immune cell recovery condition from glioma dominance (figure 3*a*). It was due to the utilization of OAA for other metabolic functions, in contrast to the glioma dominant condition, where it was released out of the system and probably used by the glioma (figure 3*b*). Upon altering the exchange of succinate and OAA from macrophages, it was observed that produced OAA in macrophages was sourced by PYDH, which completes the cycle to generate OAA (figure 3*c*). The completion of the TCA cycle through PYDH led to a decrease in the flux through FAO. As a result, glucose became a prominent energy source and was readily taken up by macrophage. This increased flux through glycolysis reduced ATP production through ATP synthase (figure 3*f*).

Additional metabolic rewiring was observed after perturbation of succinate and OAA. A snapshot of the flux distribution in glioma, T-cell and macrophage at the initiation of glioma-dominant and immune cell recovery condition from glioma dominance is provided here. It was observed that flux through glucose and glutamine uptake decreased in glioma while increasing in T-cell and macrophage. Furthermore, the release of glutamate from glioma ceased; instead, it started accumulating inside the cell. The total inhibition of glutamine synthesis and reduction in GLS are important features in reducing glioma growth [38]. Another key observation in this condition was enhanced flux through NOS and reduced flux through arginase (figure 3*d*–*f*). Downregulation of FAO, OXPHOS and arginase in T-cell and macrophage is required for the complete polarization of macrophage from an anti-inflammatory to a pro-inflammatory type as well as Treg to T-effector type [55].

#### 3.2.2. Metabolic reprogramming after blockage of glutamate uptake by T-cell

Glutamate not only plays a critical role in the development of glioma but also has an impact on T-cell functioning [10]. Previous studies explored the possibility of mitigating immunosuppression by reducing glutamate levels [9]. Blocking glutamate uptake by T-cells and its effect on the antioxidant mechanism of glioma were not studied previously.

**Figure 4.**
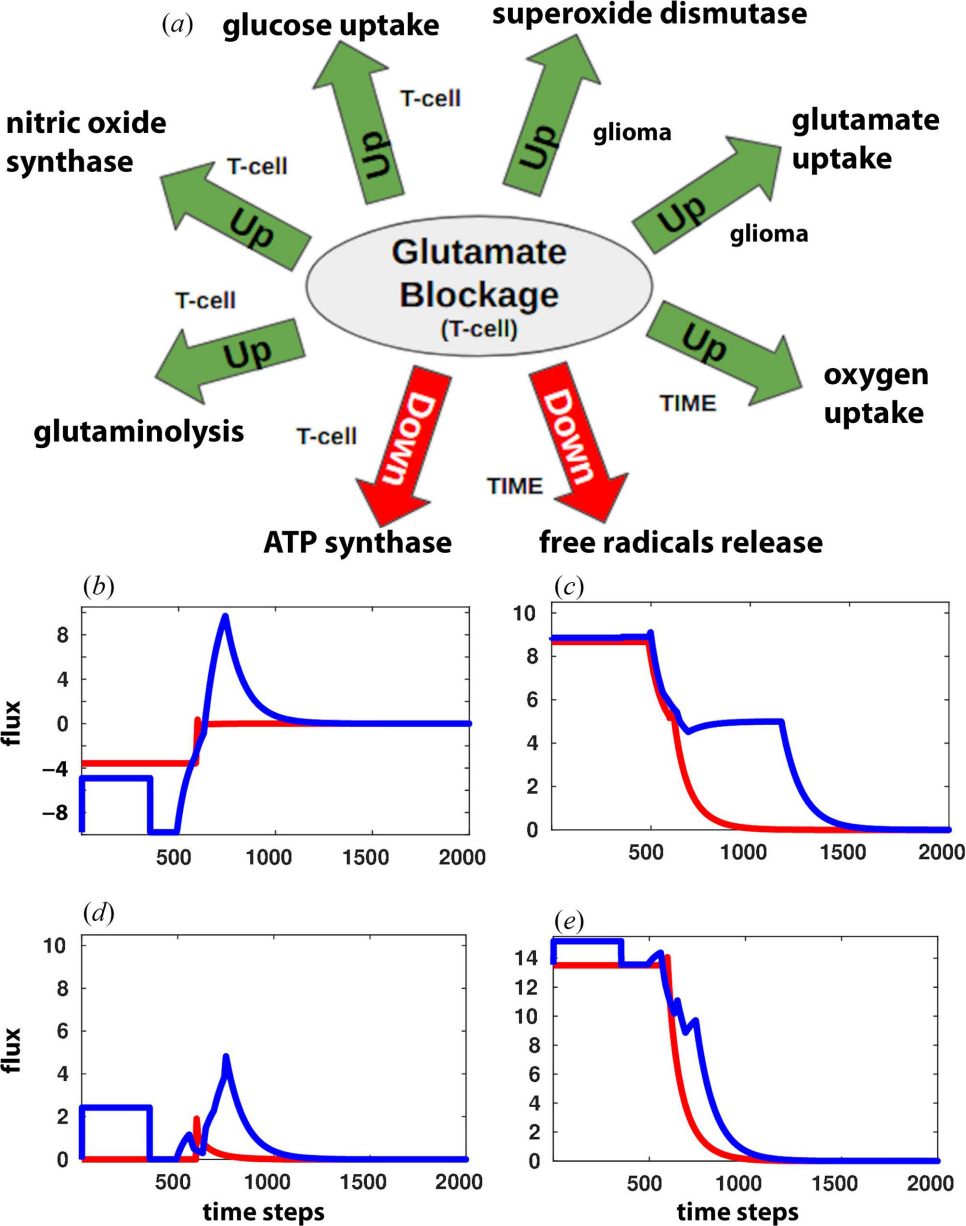
Metabolic reprogramming resulted from blockage of glutamate uptake in T-cell: (*a*) upregulated and downregulated metabolic reactions of glioma and T-cell. Temporal analysis plots: (*b*) glutamate uptake, (*c*) glucose uptake, (*d*) SOD, (*e*) oxygen release in TIME. Here, abbreviations ‘C’ and ‘T’ represent reactions in glioma and T-cells. Immune cells recovery from the glioma dominance condition (blue) is obtained after perturbation and compared with the glioma-dominant condition (red).

It was observed that blocking the glutamate uptake by T-cells led to the reprogramming of T-cell metabolism through the alteration of several important characteristics of T-cell, including increased NO synthesis, suppressed ATP production through OXPHOS, enhanced GLS and enhanced glucose and glutamine uptake by T-cell. Simultaneously, neutralization of reactive oxygen species through superoxide dismutase (SOD) and increased glutamate accumulation in glioma were also observed. Another important phenomenon observed was the increased uptake of oxygen (O_2_) in TIME, which is crucial for recovery from tumour dominance (figure 4*a*).

A temporal analysis of the flux distribution showed a significant relationship between the following four reactions: SOD activity, glutamate uptake by glioma, oxygen uptake in TIME and glucose uptake by T-cell. Upon blocking glutamate uptake to T-cell, initial release of glutamate and an increase in SOD were observed in glioma. However, over time, glutamate started accumulating and led to a repeated increase in the flux through SOD by glioma. At the same time, glucose availability to T-cells also increased (figure 4*b*–*e*). Furthermore, NO production through NOS was observed to be increased in T-cells (electronic supplementary material, figure S2*a* of file S1). However, the role of NO on tumour immunity is not clearly understood and reported to have a dual role as an immune suppressor and an activator. Activation of Th1 response was signified by changes in many other metabolic features along with an increase in NO production, such as an increase in glycolysis, glutamine uptake and glutaminolysis, and a decrease in OXPHOS and FAO (electronic supplementary material, figure S2*b–f* of file S1). These metabolic features provide strong evidence of Th1 response activation following perturbation of glutamate uptake in T-cells.

It was identified from the model that glutamate uptake blockage from T-cells causes glutamate accumulation in glioma, which further strengthens the immune response. However, the model could not identify the precise mechanism of glutamate accumulation and SOD upregulation. Future investigations are required to understand the mechanism between the blockage of glutamate uptake in T-cell, SOD activity and glutamate accumulation in glioma.

#### 3.2.3. Metabolic reprogramming through synergy of signalling and metabolism

AMPK was previously reported to play a role in maintaining glucose and fatty acid homeostasis, as well as regulating the transport of fatty acid for its oxidation [56,57]. However, its role, especially in combination with glutamate uptake, in regulating T-cell response in glioma is not defined until now. The regulation of AMPK on the transport of fatty acid for its oxidation is incorporated into the model.

The model discovers the dominant effect of AMPK regulation when combined with the inhibition of glutamate uptake in T-cells. AMPK activation triggered in response to glucose levels reaching below a critical threshold [58]. It was observed in the model that entry of fatty acid for FAO was blocked by AMPK, which enhanced the dependence of T-cell on glucose and arginine while simultaneously suppressing OXPHOS. FAO initiation occurred only when glucose reached below the critical threshold. Consequently, in the absence of FAO, glycolysis remained the primary source of energy production, resulting in an increased uptake of glucose. This, in turn, downregulated the ATP production through ATP synthase. Furthermore, in conjunction with AMPK regulation, the inhibition of glutamate uptake also enhanced the glucose and glutamine availability while downregulating OXPHOS and FAO, as observed in the previous section of the results. Consequently, the inhibition of glutamate uptake acted as an additive factor with AMPK in increasing the glucose availability to T-cells.

**Figure 5.**
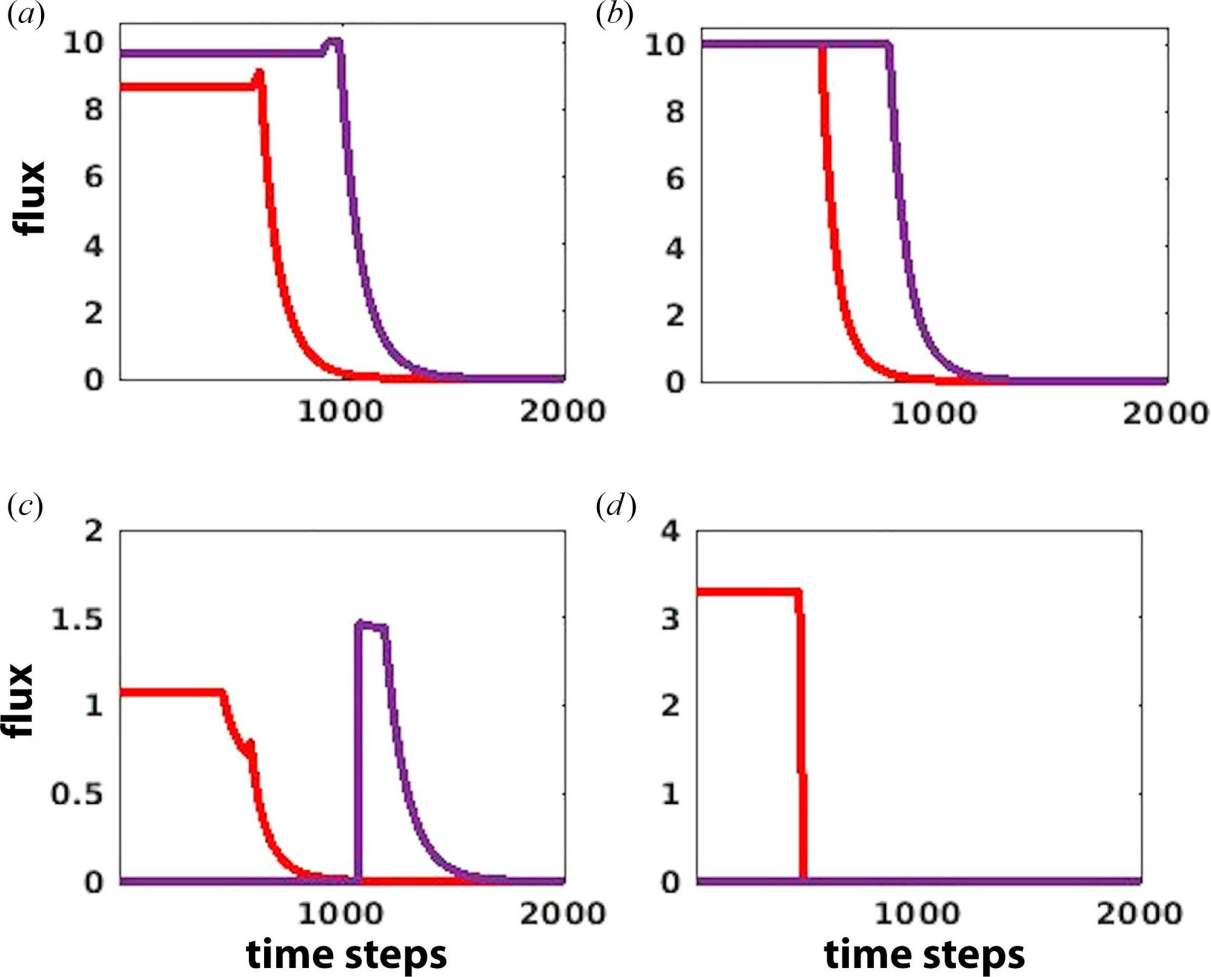
Metabolic reprogramming resulted from AMPK regulation along with the blockage of glutamate uptake and its effect on: (*a*) glucose uptake, (*b*) arginine uptake (*c*) fatty acid uptake and (*d*) ATP synthase in T-cells. Immune cells recovery from the glioma dominance condition (purple) is obtained after perturbation and compared with the glioma-dominant condition (red).

Therefore, a significant impact of AMPK, along with the blockage of glutamate uptake, was observed on OXPHOS, FAO, glucose and arginine uptake in T-cells by modulating the transport of fatty acids. This coordinated regulation resulted in an increase in glucose uptake while decreasing FAO and OXPHOS, ultimately promoting an enhanced immune effector response (figure 5*a*–*d*).

#### 3.2.4. Impact of metabolic reprogramming on immune cell gene regulatory responses

**Figure 6.**
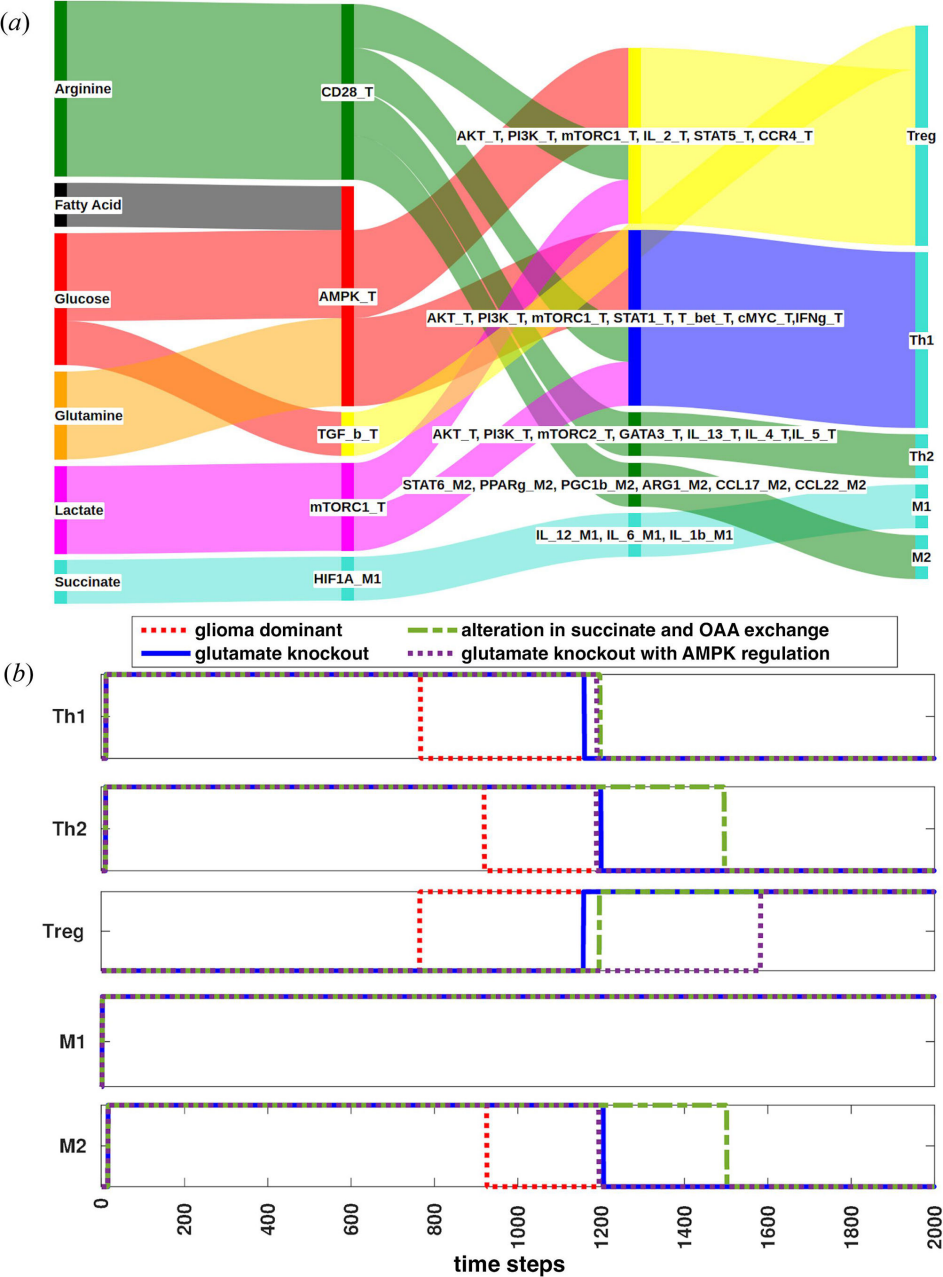
Immune responses: (*a*) T-cell and macrophage responses regulated by both signalling and metabolism, (*b*) gene regulatory immune response before and after metabolic reprogramming: in glioma-dominant condition (red), after the inhibition of glutamate uptake from T-cell (blue), alterations of succinate with OAA exchanges from macrophage (green), AMPK regulation coupled with the blockage of glutamate uptake from T-cell (purple).

The flow of metabolites between glioma, T-cell and macrophage was altered in the model to increase the pro-inflammatory response and simultaneously reduce tumour dominance. As a result, metabolic reprogramming occurred in T-cell and macrophage. The effects of metabolic reprogramming were measured on distinct phenotypes of T-cell and macrophage in the glioma-dominant and immune cell recovery conditions. Gene regulatory responses were depicted as Th1, Th2, Treg of T-cell and M1 and M2 macrophage responses (figure 6*a*).

After altering the exchange of succinate and OAA between glioma and macrophage, it was observed that the Th1 response was increased while the Treg response was delayed. This effect was due to the increased availability of glucose, glutamine and arginine coupled with reduced availability of lactate to T-cells. However, the increased availability of arginine, along with glucose, led to an elevation in Th2 and M2 responses too. The M1 response remained constant due to the constant availability of succinate. Similarly, the inhibition of glutamate uptake by T-cell also increased Th1 and reduced the Treg response (figure 6*b*). The observed gene regulatory response in this condition was suboptimal because glucose and arginine availability did not increase as much as in the previous condition.

Notably, it was consistently observed in previous perturbations that Th2 and M2 response was always more than Th1, primarily due to increased arginine availability, as the dependence of Th2 and M2 response was solely on the arginine metabolite. Furthermore, our analysis revealed that a combination of signalling (AMPK) and metabolic (glutamate) targets effectively triggered the Th1 response more than Th2 and delayed the activation of the Treg response to the maximum. To promote Th1 response over Th2 and M2 responses, it was necessary to maintain arginine availability lower than glucose. When arginine availability exceeded that of glucose, Th2 and M2 responses dominated. The combination of signalling and metabolic targets was the only condition where Th1 response was more than Th2.

It was observed from the model that an increase in Th1 and a decrease in Th2, M2 and Treg responses resulted from increased availability of glucose, glutamine, reduced availability of lactate and arginine availability being less than that of glucose to T-cell (figure 6*b*). Therefore, to achieve an optimal immune response, it was necessary to increase the availability of glucose, glutamine, reduce the availability of lactate and arginine availability lower than that of glucose.

The increase in Th1 and the decrease in Treg response following the combination of signalling and metabolic target alteration emerged as intriguing observations. This suggests that with glucose and arginine metabolites available to immune cells at defined levels, the Th1 response dominated and suppressed the tumour response for quite an extended period. However, immune cells appeared to switch their effector response once the metabolite concentration dropped below the threshold level. This led to an upregulation of the Th2, Treg and the anti-inflammatory response of macrophage. The transition in immune cell behaviour highlights the complex and dynamic interplay between signalling and metabolism factors in regulating the immune response against glioma.

#### 3.2.5. Comprehensive insights into immune cell functions through holistic metabolism

This is the first holistic model describing the significance of immunometabolism in determining the phenotypic characteristics and functions of T-cell and macrophage in TIME. By incorporating the necessary and known metabolic alterations in glioma and astrocyte, distinct metabolic characteristics of T-cell and macrophage in normal astrocyte and glioma-dominant conditions were obtained. The combined impact of these alterations in determining the immune cell phenotypes was interesting to achieve, for example, Treg and M2 type in glioma-dominant condition. Thereby, these alterations can also be helpful in generating the exhausted T-cell and macrophage scenario.

Immune cell-specific metabolic properties in glioma-dominant condition were observed to be immunosuppressive. Furthermore, the model identifies metabolic targets that reprogram the metabolism of T-cell and macrophage and favour anti-tumour immune responses. Metabolic reprogramming through modulation either through exchanges or blockage of metabolites shifted the immune response towards pro-inflammatory. The effect of reprogrammed metabolism on T-cell and macrophage gene expression response revealed the shift of T-cell and macrophage phenotype from immunosuppressive to immunostimulant. Successful reprogramming of the metabolism of T-cell and macrophage and observing the effect of the holistic metabolic changes on gene expression provide a system-level comprehensive view of immune cell functions.

The key accomplishment of the model lies in its ability to capture the dynamic transitions of T-cell and macrophage phenotypes through metabolic reprogramming in glioma-dominant and recovery conditions. In glioma-dominant condition, there was dominance of Th2, Treg and M2 phenotypes. However, through metabolic reprogramming, these responses were reduced while Th1 was increased. Dynamic regulations of metabolism on immune cell-specific gene expression response provides a comprehensive assessment of Th1, Th2, Treg, M1 and M2 responses at the system level. Integrating the metabolic characteristics of immune cells with glioma and simultaneously measuring the gene expression response will undoubtedly lead to new efficacious therapies for cancer patients.

## 4. Discussion

Tumour and immune cells, being co-existent, impose regulations on each other. Due to the continuous and rapid progression of the tumour, the availability of key nutrients is skewed towards the tumour. The scarcity of nutrients, accumulation of specific metabolites, generation of by-products, etc. can impair activation and differentiation of immune cells necessary for mounting effective anti-tumour response. Recently, immunoediting through immunometabolism has gained a lot of attention [59,60]. So far, the development of tumour–immune interaction models by integrating signalling and metabolic pathways is limited and needs further exploration.

To understand the complex tumour–immune interactions, a comprehensive system-level model is developed through incorporation of signalling and metabolic pathways of glioma, T-cell and macrophage, along with their regulatory interactions within and between cells. A network is reconstructed by integrating T-cell, macrophage, astrocytes and glioma-specific pathways. Importantly, cross-talks of signalling and metabolism between and within cells are included in the model. Dynamic interactions between metabolism, signalling and the gene regulation are incorporated by rFBA, which integrates Boolean- and FBA-modelling approaches.

This study provides pioneering insight into the influence of metabolism on gene regulatory immune response along with the subsequent regulatory constraint imposed on metabolism. It must be noted that signalling and metabolic systems are integrated at different time scales, and the acquired time scales correctly validate the behaviour of T-cell, macrophage and glioma in TIME. This study suggests the immune-modulatory mechanisms capable of shifting anti-inflammatory responses towards pro-inflammatory responses against glioma, by altering the flow of metabolites between glioma and immune cells.

The model analysis predicted potential metabolites or combinations of metabolites to modulate the immune response in TIME. The notable finding of the model was the identification of a combination of succinate and OAA. Modifying the flow of these metabolites between macrophage and glioma shifted the metabolism towards pro-inflammatory response and increased the availability of glucose and arginine while simultaneously decreasing OXPHOS and FAO. Reduced fatty acid dependence and simultaneously increased glucose uptake in macrophages were due to the completion of the TCA cycle through PYDH. Significantly, this combination also modulated the glioma-specific metabolic properties, leading to the suppression of glioma. Another significant observation of the model was that the inhibition of glutamate uptake by T-cell reprogrammed the antioxidant mechanism of glioma and also increased the availability of glucose and arginine with the reduction in OXPHOS and FAO. This, in turn, enhanced the immune effector response against glioma. With disruption in the antioxidant mechanism of glioma, an increase of O_2_ in TIME, downregulation of OXPHOS, upregulation of GLS, glucose and glutamine uptake in T-cell, accumulation of glutamate in glioma shifted T-cell metabolism towards Th1.

The most significant immunostimulation was achieved after combining signalling and metabolic targets. It was observed in the model that the regulation of AMPK coupled with the blockage of glutamate uptake from T-cell increased the availability of glucose and arginine by regulating fatty acid transport. In the model, Th2 and M2 responses were particularly dependent on arginine levels. Through the combination of signalling and metabolic targets, arginine availability was maintained lower than that of glucose which allowed for a greater Th1 response than Th2. Furthermore, the activation of Treg response was delayed compared with previous conditions. Notably, AMPK was discovered to be the signalling molecule in the model that affects the consumption rate of glucose and arginine. A combination of signalling and metabolic targets (AMPK and glutamate) was identified as the most effective approach in enhancing the immune effector response.

The designed model has both advantages and limitations. Perturbation analysis was performed in either T-cell or macrophage; it could also be developed in tumour cells. This model measured enhanced immune cell behaviour to overcome tumour-dominant conditions, and the expression profile of glioma-specific genes was not measured following perturbations. Subcellular localization and compartmentalization were not considered in the metabolic network. Some of the mechanisms in the model could not be thoroughly understood and require future investigations—for example, the role of arginine metabolism in regulating Th1, Th2 and M2 response. Arginine regulates T-cell response through the co-receptor CD28, affecting both Th1 and Th2 responses. To acquire differential Th1 and Th2 responses, reconsidering the regulation of arginine metabolism on immune cells is required to study further. Next, the mechanism of SOD from glioma after blocking the glutamate uptake in T-cell requires further investigation.

The key reason to develop this model was to drive advancement in cancer treatment by developing more effective therapeutic strategies. The designed model predicted promising targets for anti-tumour therapy by modulating the immune response against glioma, such as the combination of succinate and OAA, which helped in tumour recovery through macrophage polarization. Furthermore, the inhibition of glutamate uptake in T-cell was also identified as an effective target in boosting immune response by disrupting the antioxidant metabolism of glioma. However, the most effective identified targets were the combination of signalling (AMPK) and metabolic (glutamate) targets, which significantly enhanced the immune–effector response while reducing the suppressive response of immune cells. Determined targets from the model can be exploited as possible immunomodulators. Targeting these may circumvent the inhibitory effects of glioma dominance and strengthen the anti-tumour immune responses. Therefore, this study advances the understanding of glioma–immune interaction, specifically through signalling and metabolic cross-talks. Additionally, it also identifies a combination of metabolic as well as the combination of signalling and metabolic targets that have the potential to guide immune cells in combating glioma. Identification of these targets for combination therapy is the future area of focus. Synergistic potential of targeting both signalling molecules and metabolites holds promise for revolutionizing treatment strategies. As a next step, the goal is to transform the model into a pipeline that can seamlessly incorporate additional spatial transcriptomics data from the tumour microenvironment. This pipeline will assist clinicians in pinpointing optimal combinations of signalling and metabolic targets to amplify the immune response of macrophages (focusing on M1 properties) by considering the specific characteristics of the patient’s tumour microenvironment. Moreover, this approach is versatile and adaptable, easily applicable to various tumour types, and their respective T-cells and macrophages, allowing the customization of personalized immunotherapy treatments.

## Data Availability

All data are provided in the electronic supplementary material: supplementary_file1 (file S1), supplementary_file2 (file S2) and supplementary_file3 (file S3) [61].
